# Use of Humanised Rat Basophilic Leukaemia Cell Line RS-ATL8 for the Assessment of Allergenicity of *Schistosoma mansoni* Proteins

**DOI:** 10.1371/journal.pntd.0003124

**Published:** 2014-09-25

**Authors:** Daniel Wan, Fernanda Ludolf, Daniel G. W. Alanine, Owen Stretton, Eman Ali Ali, Nafal Al-Barwary, Xiaowei Wang, Michael J. Doenhoff, Adriano Mari, Colin M. Fitzsimmons, David W. Dunne, Ryosuke Nakamura, Guilherme C. Oliveira, Marcos J. C. Alcocer, Franco H. Falcone

**Affiliations:** 1 Division of Molecular and Cellular Science, School of Pharmacy, University of Nottingham, Nottingham, United Kingdom; 2 School of Biosciences, University of Nottingham, Sutton Bonington Campus, Loughborough, United Kingdom; 3 Genomics and Computational Biology Group, Centro de Pesquisas René Rachou, National Institute of Science and Technology in Tropical Diseases, Fundação Oswaldo Cruz - FIOCRUZ, Belo Horizonte, Minas Gerais, Brazil; 4 School of Life Sciences, University of Nottingham, Nottingham, United Kingdom; 5 Center for Molecular Allergology, IDI-IRCCS, Rome, Italy; 6 Associated Centres for Molecular Allergology, Rome, Italy; 7 Department of Pathology, University of Cambridge, Cambridge, United Kingdom; 8 Division of Medicinal Safety Science, National Institute of Health Sciences, Setagaya-ku, Tokyo, Japan; University of Cambridge, United Kingdom

## Abstract

**Background:**

Parasite-specific IgE is thought to correlate with protection against *Schistosoma mansoni* infection or re-infection. Only a few molecular targets of the IgE response in *S. mansoni* infection have been characterised. A better insight into the basic mechanisms of anti-parasite immunity could be gained from a genome-wide characterisation of such *S. mansoni* allergens. This would have repercussions on our understanding of allergy and the development of safe and efficacious vaccinations against helminthic parasites.

**Methodology/Principal Findings:**

A complete medium- to high-throughput amenable workflow, including important quality controls, is described, which enables the rapid translation of *S. mansoni* proteins using wheat germ lysate and subsequent assessment of potential allergenicity with a humanised Rat Basophilic Leukemia (RBL) reporter cell line. Cell-free translation is completed within 90 minutes, generating sufficient amounts of parasitic protein for rapid screening of allergenicity without any need for purification. Antigenic integrity is demonstrated using Western Blotting. After overnight incubation with infected individuals' serum, the RS-ATL8 reporter cell line is challenged with the complete wheat germ translation mixture and Luciferase activity measured, reporting cellular activation by the suspected allergen. The suitability of this system for characterization of novel *S. mansoni* allergens is demonstrated using well characterised plant and parasitic allergens such as Par j 2, SmTAL-1 and the IgE binding factor IPSE/alpha-1, expressed in wheat germ lysates and/or *E. coli*. SmTAL-1, but not SmTAL2 (used as a negative control), was able to activate the basophil reporter cell line.

**Conclusion/Significance:**

This method offers an accessible way for assessment of potential allergenicity of anti-helminthic vaccine candidates and is suitable for medium- to high-throughput studies using infected individual sera. It is also suitable for the study of the basis of allergenicity of helminthic proteins.

## Author Summary

Infection with parasitic helminths is characterised by a marked elevation of total and parasite-specific Immunoglobulin E (IgE). It is widely believed that this IgE response has evolved to protect hosts against large metazoan parasites. Such a protective function has been well characterised in particular against members of the genus *Schistosoma*. However, with a few notable exceptions, the molecular targets of the IgE response and the downstream immunological mechanisms leading to host protection are not well understood. The molecular targets of a specific IgE response are by definition called allergens. While almost 3,000 different allergens, contained in e.g. plant pollen or seeds, moulds or animal materials, have been characterised at the molecular level, and are listed and described in databases such as the Allergome database (www.allergome.org), only a few dozen allergens have been characterised in parasitic helminths. A more detailed understanding of the molecular targets of the anti-helminth IgE response can not only be expected to further our basic understanding of protective immune responses and allergy in general–such knowledge can also be expected to have important repercussions on the production of safe and effective anti-helminthic vaccines. This research describes a novel approach suitable for genome-wide functional identification of allergens in *S. mansoni* and other parasites, paving the way for the identification of the *Schistosoma* allergome.

## Introduction

Helminthic parasites are well known to induce a strong Th2-biased response in their hosts, characterised by elevated levels of total and parasite-specific IgE, IL-4, IL-5 and IL-13, with concomitant expansion and mobilization of specific effector cells [1], [2]. This IgE response is widely believed to have evolved to protect against ectoparasites and parasitic helminths, and *Schistosoma* in particular [3], although this widespread view has been recently challenged [4].

Human infection with the trematode *Schistosoma mansoni* is well known to correlate with a progressive increase of serum IgE levels [5]. *S. mansoni* infection usually peaks in early adolescence and declines in adulthood, a pattern that suggests that individuals in endemic areas can gradually acquire an age-related resistance to reinfection [6], [7]. Progressive acquisition of anti-schistosome immunity coincides with natural death of worms (averaging 10–15 years of life), an event during which the parasites release and expose previously inaccessible antigens to the immune system [8]. Similarly, repeated treatment with praziquantel can speed up the process of immunity, resulting (in some individuals) in so-called post-treatment resistance to infection [7], [9]–[11]. A Th1-type or mixed Th-1/Th2-type response is associated with putative natural resistance in ‘endemic normal’ individuals [12]. However, post-treatment resistance is associated with a stronger Th2-type response dominated by IgE and IgG4 [5], with the higher IgE/IgG4 ratio, rather than their absolute levels, best predicting resistance [13]–[15]. A group of antigens related to the different infection status of endemic area residents in Brazil was recently identified by a serological proteomic analysis which may be related to susceptibility or resistance to infection [14]. However, despite recent progress and decades of research, the targets of this protective antibody response and the basis of its ‘inefficient’ acquisition are still unknown.

The occurrence of natural and post-treatment resistance suggests that immunity could be conferred by appropriately formulated vaccines, possibly using mixtures of antigens. Strategies used for vaccine development have changed as the genomic data for schistosomes have become increasingly available and post-genomic technologies have matured [15]. The traditional approach has been to identify immunogenic antigens using immunological screening (i.e. Western Blots), followed by cloning, expression and case-by-case testing for protection in murine or other animal models. To date, even the best vaccine candidates have achieved protection levels below 70% in animal models, with higher protection only achieved by using high doses of irradiated cercariae [16], [17]. The most promising vaccine candidate (SmTSP-2) achieved 57% and 64% reduction (adult worm and egg burden, respectively) and importantly was recognized by IgG1 and IgG3, but not IgE, in sera of naturally resistant, but not uninfected or chronically infected individuals [18].

There is however a major unsolved conundrum specific to the development of anti-helminthic vaccines. While the bulk of the evidence points to a major protective role of the parasite-specific IgE response against the parasite, vaccinating with an allergen bears the inherent risk of potentially inducing hazardous allergic reactions in sensitised individuals, as recently reported during clinical trials for an anti-hookworm vaccine using Na-ASP-2, where adult volunteers experienced generalized urticarial reactions immediately after vaccination [19]. It could be shown that individuals who displayed urticarial reactions possessed high levels of IgE against Na-ASP-2. This led to testing of specific IgE levels for other candidate vaccine antigens such as *Necator americanus* GST1 and APR1 using sera from individuals resident in helminth-endemic areas [20]. Thus it would be beneficial to identify such allergens at an early stage during vaccine development.

We have previously shown that human basophils are sensitised within 6 weeks of a single, low-dose infection with *N. americanus* infective stage larvae [21]. Basophil activation could be detected by flow cytometry in the absence of measurable parasite-specific IgE levels in the serum. This suggests that basophils may offer a sensitive way of measuring the presence of parasite antigen-specific IgE in infected individuals, and, perhaps more importantly in the context of vaccination, to demonstrate the ability of allergens to induce basophil or mast cell activation, in contrast to measuring allergen binding by specific IgE alone. Therefore, we recently developed a new detection system for antigen-specific IgE based on the NFAT-dependent luciferase expression in a humanised rat basophilic leukaemia cell line (RS-ATL8) [22], [23]. When sensitised with egg white-allergic patient's serum, this cell line detected at least 1 fg/mL of egg white extract proteins as a luciferase expression [23].

The sensitivity of this detection method makes it possible to study the potential allergenicity of a protein using only minute amounts of protein. The lack of requirement for a high yield allows the use of a fast and easy cell-free expression system such as wheat germ lysate, which allows for expression of microgram amounts of protein [24] in less than two hours. This in turn makes it possible to produce many correctly folded antigens of interest in short time [25].

Here, we demonstrate proof-of-principle of how the RS-ATL8 cell line, in combination with a cell-free *in vitro* translation system and a set of stringent quality controls, can be used for assessment of allergenicity of *S. mansoni* antigens. This technology paves the way for high-throughput, genome-wide assessment of *S. mansoni* antigen allergenicity - the *Schistosoma* allergome.

## Materials and Methods

### Human sera

Samples of schistosomiasis patients were from a Ugandan study. All EDTA plasma samples were obtained from a male cohort from the village of Musoli on Lake Victoria. Samples used in this study are from a subgroup of infected people described in Fitzsimmons *et al.* [26]. The blood samples were selected based on their known content of SmTAL1-specific IgE as measured by isotype specific ELISA, as described by Naus and co-authors [27]. Details of the plasma samples are summarised in Table S1 in the Supplementary data.

Ethical clearance was obtained from the Uganda National Council of Science and Technology (ethics committee for Vector Control Division, Ugandan Ministry of Health) who approved the age of consent as 15 y at the time of sample collection (2004/2005). Consent forms were translated into the local language and informed written consent was obtained from all adults and from the parents/legal guardians of all children. Parental consent was not sought for individuals 15–18 y old.

Sera from patients allergic to *Parietaria judaica* (commonly known as spreading pellitory in the Mediterranean area, and sticky weed or asthma weed in Australia) were collected after informed consent from the patients, and under a study protocol approved by the institutional ethical committee to establish the sera bank. Institutional Review Board of IDI-IRCCS, Rome, Italy (n. 106-CE-2005). The sera obtained from nine patients were pooled in equal amounts and the levels of specific IgE, IgG4, and total IgG against 104 103 different allergens measured by ImmunoCAP ISAC multiplexing analysis (ThermoScientific) following the protocol previously described [28]. The pooled sera showed high levels of specific IgE to Par j 2 (55 U/ml). The complete ISAC characterisation of the pooled sera is shown in the Supplementary data (Table S2).

#### Viral inactivation of plasma or serum

To reduce biohazard of the blood samples, which otherwise would require the work to be performed in laboratories with increased biological safety levels, all acellular blood samples were subjected to a detergent treatment known to inactivate enveloped viruses in serum or plasma samples [29] and has been used for IgE testing by Poulsen and Sørensen [30]. Sera were incubated with a mixture of 0.3% (v/v) TNBP (Tri-N-butylphosphate) and 1% (v/v) Tween-80 (Polyoxyethyleneorbitan) under rotation at room temperature for 1 hr, aliquoted and frozen until further use.

### Polymerase chain reaction (PCR)

Fifteen chosen genes representing proteins from a diverse families such as major egg allergens, troponin, fatty acid binding protein (complete list described in supplementary data in Table S3) were amplified from relevant cDNA libraries (adult worm λZAP cDNA library generous donation by K. Hancock, CDC Atlanta, USA; egg stage λZAP cDNA library kindly contributed by Helmut Haas and Gabi Schramm, Research Centre Borstel, Germany), or available cDNA clones (kindly donated by Alan Wilson, University of York, UK) using 25 µL JumpStart REDTaq ReadyMix Reaction Mix (Sigma-Aldrich), 2.5 µL of each forward and reverse custom made gene specific primer (Sigma Aldrich, final concentration 0.5 µM), 2 µL of *S. mansoni* cDNA library in 50 µL final volume. For longer sequences, Q-5 polymerase (New England Biolabs) was used to take advantage of its high proofreading activity. Forward primers were constructed by adding a SgfI restriction site and a start ATG (where not available, i.e. when expressing the mature protein sequence after leader peptide cleavage at the 5′ end), and reverse primers by adding a His_6_-tag followed by a valine (for facilitation of subcloning into other expression vectors) and a stop codon, followed by a PmeI restriction site at the 3′ end. The complete primer sequences are listed in Table S3 in Supplementary materials.

Temperature gradients were run initially to optimise the annealing temperature for the different genes; however an annealing temperature of 54°C worked for most of the genes. Successful PCR was confirmed by 1% agarose gel electrophoresis, using 100 bp Tridye DNA ladder (New England Biolabs) for reference. The final products were purified using a Promega Wizard SV Gel and PCR Clean-Up System, as described by the manufacturer.

### Cloning and transformation

The concentration of the amplified genes was measured using a NanoDrop 1000 Spectrophotometer (Thermo Scientific). The purified PCR products were inserted into pF3A WG (BYDV) Flexi Vector (Promega) using the Flexi Vector System (Promega). The ligations were heated at 65°C for 5 min for T4 DNA ligase (HC) inactivation, before transformation, which substantially increased the number of colonies obtained. Transformation was achieved by employing electro-competent DH5α *Escherichia coli* cells using a BioRad Micropulser electroporation apparatus, following standard molecular biology procedures. Plasmids were purified from the transformed cells using a QIAGEN QIAprep Spin Miniprep Kit, as per the manufacturer's protocol. All purified plasmids were verified by DNA sequencing (Source Bioscience, Nottingham, UK).

### Coupled *in vitro* transcription-translation

Plasmids were used to produce proteins through coupled *in vitro* transcription-translation, using TnT SP6 High-Yield Wheat Germ Protein Expression System (Promega), as per the manufacturer's instructions. Protein synthesis was initiated by mixing the appropriate DNA template (2–3 µg), 30 µL of the TnT SP6 High-Yield Wheat Germ Master Mix and water for a 50 µL final volume, and then incubating the reaction at 25°C for 2 hours. Protein expression was analyzed by the incorporation of labelled lysine residues (FluoroTect GreenLys, Promega) in a 10 µl aliquot of the plasmid/wheat germ lysate mixture (WGL) as directed in the instructions. Samples were heated for 3 minutes at 70°C, run on 4–20% SDS-PAGE gradient gels (BioRad, UK), under reducing conditions and imaged with a laser-based fluorescent gel scanner (Fujifilm LAS-4000 319 Imaging System). Molecular weights (MW were estimated using the Kaleidoscope Precision plus marker (BioRad, UK) which contains several fluorescently labelled components.

### Recombinant bacterial expression of Sm-TALs

SmTAL1 and Sm-TAL2 were expressed in E. coli as described previously [31].

### Western blots

IPSE/alpha-1, SmTAL1, and SmTAL2 proteins produced in the wheat germ lysate system were transferred to a 0.45 µm nitrocellulose membrane (NCM) (Sigma-Aldrich) and sections of membrane were incubated with mouse anti-IPSE/alpha-1 monoclonal antibody (1∶2000) or rabbit anti-sera (1∶500) against SmTAL1 or SmTAL2, using the method described by Burnette [32]. The anti-IPSE/alpha-1 monoclonal antibody used is from clone 74 2G4 [33], [34]which recognises both monomeric and dimeric IPSE/alpha-1. Both antibodies were diluted in TBS with 3% Tween, 30% Wheat Germ Extract and 5% skimmed milk powder.

The mouse or rabbit primary antibodies were detected with an HRP-conjugated secondary antibody using chemiluminescence (ECL Plus Western Blotting Detection System, GE Healthcare) diluted 1∶5000 and visualised using a Fujifilm LAS-4000 imaging system.

### Cell culture and measurement of basophil activation

RS-ATL8 cells were cultured in 75 cm^2^ flasks, with 0.2 µm vent caps (Corning, USA), in an incubator set at 37°C with 5% carbon dioxide with a humidified atmosphere [22], [23]. The flasks contained 10 mL MEM (GIBCO, USA), supplemented with 5% v/v heat-inactivated FCS (GIBCO, USA), 100 U/mL penicillin, 100 µg/mL streptomycin (Sigma, UK) and 2 mM L-glutamine (Sigma, UK), with medium change every 2–3 days. Cells were detached by washing the flasks twice with calcium/magnesium-free DPBS, followed by incubation with 2 mL trypsin-EDTA (GIBCO, USA) for 10 minutes. Alternatively, cells were scraped using cell scrapers (TPP, Switzerland). 1 mg/mL G418 (Fisher ThermoScientific, UK) and 600 µg/mL hygromycin B (Invitrogen, Paisley, UK) were used to maintain expression of human FcεRI genes and NFAT-luciferase, respectively. Prior to testing, cells were incubated overnight in culture medium with various dilutions of pooled serum from *Parietaria judaica* patients or *S. mansoni* infected individuals, and washed once prior to addition of the stimulus (recombinant Par j 2 from Bial, Zamudio, Spain, accession: R-17). The following positive control was used for all RS-ATL8 experiments: sensitization with 1 µg/mL of human IgE (AbD Serotec) followed by stimulation with 1 µg/mL of goat anti-human IgE polyclonal IgG (Vector Labs).

ONE-Glo Luciferase Assay System (Promega, UK) was used for all luciferase assays, following the manufacturer's instructions. Half volume (50 µL) reactions were used. Chemiluminescence was measured on an Infinite M200 microplate reader (Tecan, Männedorf, Switzerland) not later than 30 minutes after the addition of the luciferase substrate.

### Statistical analysis

One-way ANOVA followed by Dunnett's or Tukey's post hoc test was performed using GraphPad Prism 6 software for Figures 3, 4, 6, 7. Spearman's rank correlation test was performed to compare IgE titres with the luminescence response in activated RS-ATL8 cells (Figure 8).

## Results

**Figure 1:**
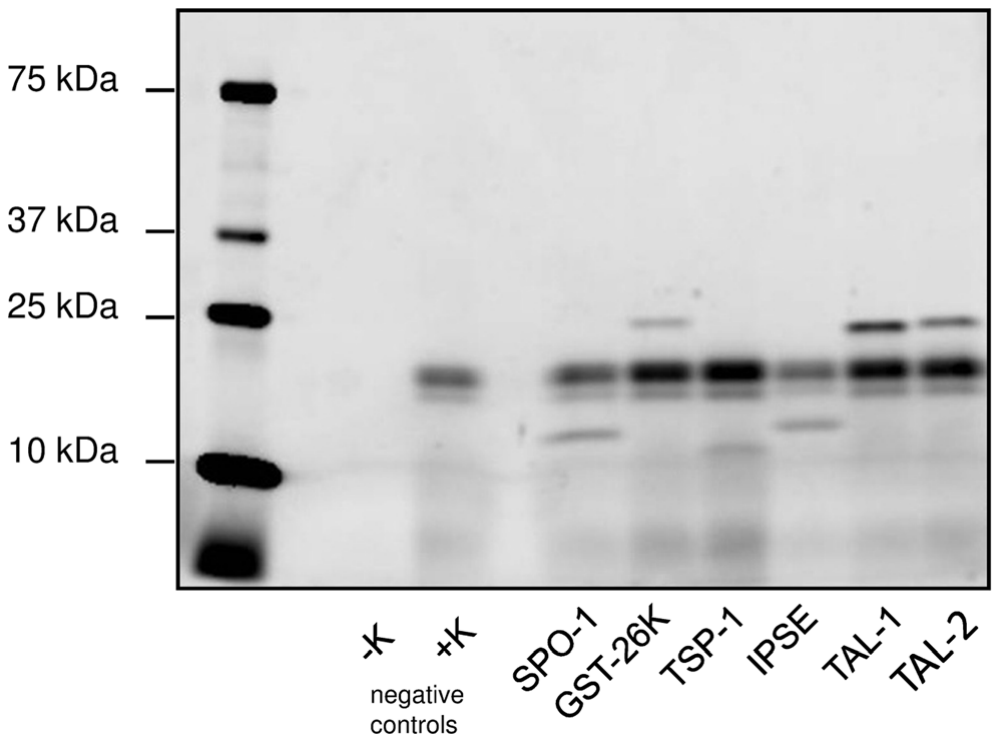
In gel detection of five different *S. mansoni* antigens expressed in vitro using WGL. SPO-1: Smp_113760; GST-26k: Smp_102070; TSP-1, extracellular loop 2: Smp_095630; IPSE/alpha-1: Smp_112110; SmTAL1: Smp_045200; SmTAL2: Smp_086480. Details of sequences and expected molecular weights are given in the Supplementary Data in Table S3. Success of translation was monitored by incorporation of BODIPY-labelled fluorescent Lysine in separate aliquots during translation. Samples were run on 4–20% SDS-PAGE gradient gels under reducing conditions and imaged in a Fujifilm LAS-4000. The left lanes show the wheat germ lysate control without template DNA, either without (−K) or with Lysine incorporation (+K), indicating fluorescent components produced during *in vitro* translation from endogenous mRNA.

All the genes described herein were successfully amplified and ligated into the p3FA (BYDV) WG vector. The annealing temperature of 54°C was efficient for all the genes and DNA sequences were confirmed for the clones before *in vitro* expression. All fifteen reported parasitic genes reported herein were then successfully translated *in vitro* using the coupled transcription/translation WGL mixture and an aliquot of the translation mix was used for monitoring of translation by incorporation of fluorescently-tagged lysine. An example of 5 genes obtained by this method is shown in Figure 1. As seen in the negative control, protein translation with WGL results in two endogenous fluorescent components in the 15–20 kDa range (lane three in Fig. 1) which have the potential to interfere with detection of the translated parasitic protein if of this size. More examples can be seen in Supplementary data Figure S1.

**Figure 2:**
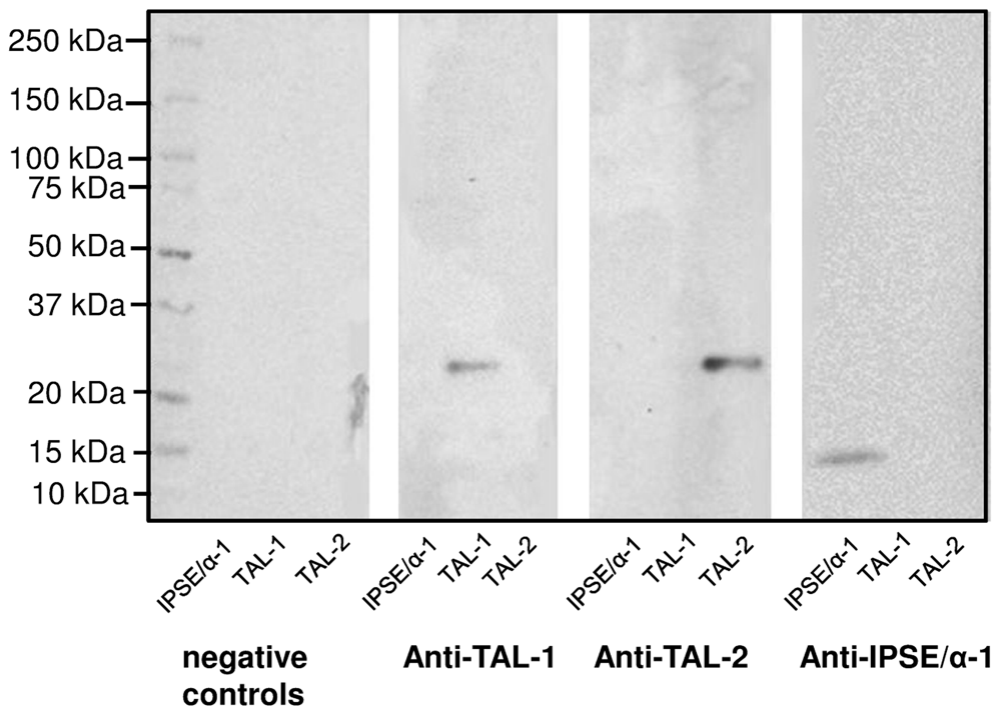
Western blot demonstrating antigenicity of the *S. mansoni* proteins expressed in the WGL system. Non-purified wheat germ extracts containing *in vitro* translated IPSE/alpha-1, SmTAL1 or SmTAL2 were separated in a 4–20% SDS-PAGE gel and blotted onto NCM. Separate strips of NCM were treated with anti-TAL1 rabbit serum, anti-TAL2 rabbit serum or anti-IPSE/alpha-1 mouse monoclonal antibody. The negative control (neg. control) was incubated without primary antibody/serum, but with secondary antibody. Membranes were imaged using chemiluminescence and a Fujifilm LAS-4000.

In order to assess whether cell-free translation in wheat germ lysates results in proteins with unaltered antigenic properties, three unlabelled *S. mansoni* genes (IPSE/alpha-1, SmTAL1, SmTAL2), for which either polyclonal antisera or monoclonal antibodies were available, were expressed using wheat germ lysates and tested by immunoblotting. As shown in Figure 2, all three antigens were specifically recognised by the corresponding antibodies, demonstrating that the method chosen for expression does not appear to alter antigenicity of the parasitic proteins.

**Figure 3:**
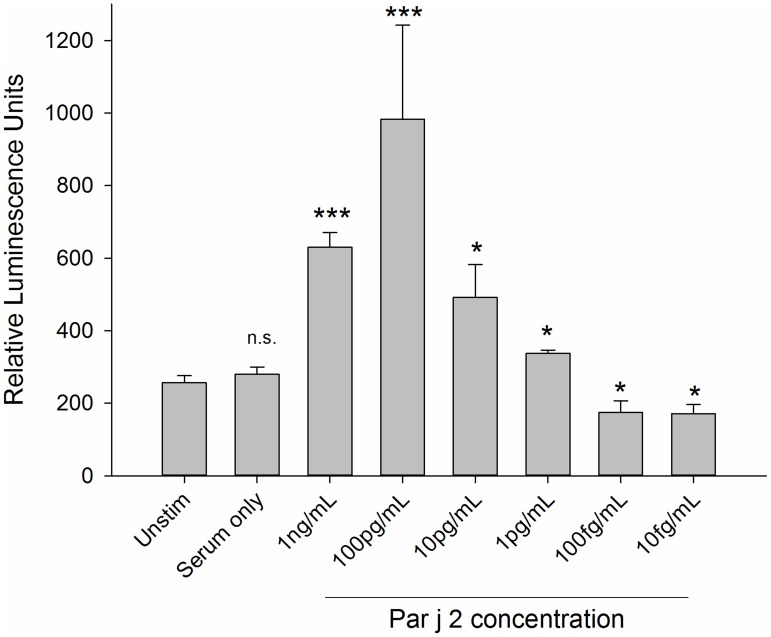
Optimal concentration and minimal concentration of detectable allergen. This was determined by sensitising RS-ATL8 cells with the pooled serum (1∶50 dilution) from individuals with a specific IgE response to the Par j 2 allergen and challenged with recombinant Par j 2, serially diluted from 1 ng/mL to 10 fg/mL in 1∶10 dilution increments. Data are mean ±SD of the readings of three separate wells. Multiplicity adjusted p-values (ANOVA followed by Dunnett's post-hoc test) ***: *p*<0.001, *: *p*<0.05, n.s.: not significant. Representative of three separate experiments performed in triplicates with comparable results.

**Figure 4:**
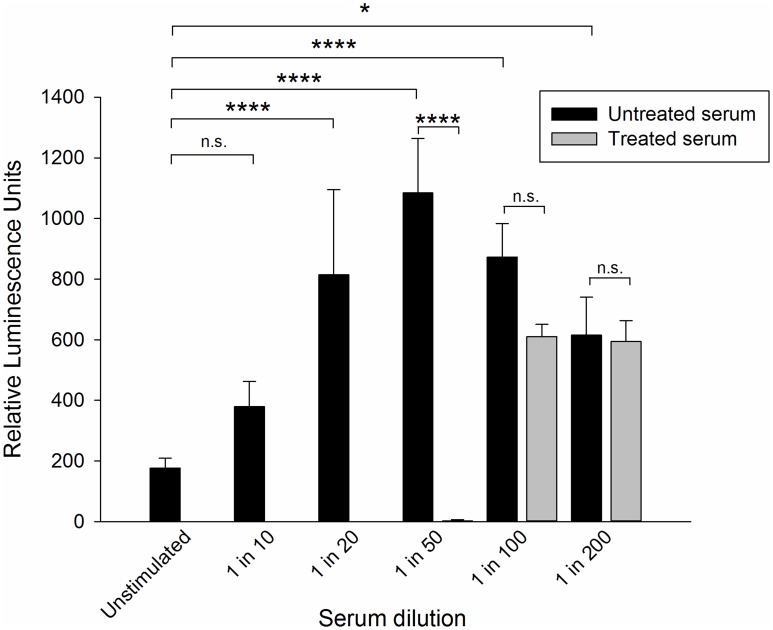
Luminescence data from the determination of serum-mediated and detergent-mediated cytotoxicity. The Par j 2 serum pool was incubated with a mixture of 0.3% (v/v) TNBP (Tri-N-butylphosphate) and 1% (v/v) Tween-80 (Polyoxyethyleneorbitan) and incubated under rotation at room temperature for 1 hr. Treated and untreated serum samples were then used in the indicated dilutions (v/v) to sensitise RS ATL8 cells overnight, followed by stimulation with 100 pg/mL Par j 2 recombinant allergen the next day. Data are mean ±SD of the readings of three separate wells. Positive and negative controls included in this experiment (A23187 and PMA: 1840.00±168.02 RLU; IgE+anti-IgE: 4991.00±171.62 RLU) are not shown. N.s.: not significant; n.t.: not tested; *: p<0.05; ****: *p*<0.0001 (ANOVA followed by Tukey post-hoc test, all p-values adjusted for multiplicity).

Initial experiments using cells sensitised with human monoclonal myeloma IgE (with unknown specificity) overnight and stimulated with an anti-human IgE antibody had demonstrated a high sensitivity of 10 pg IgE per assay (Supplementary Figure S2). To determine the sensitivity of the assay using polyclonal IgE in serum, a dose response curve of RS-ATL8 cell sensitised overnight with pooled sera diluted 1∶50 obtained from *Parietaria judaica* allergic patients, stimulated with the matching allergen Par j 2, was performed. These experiments showed that activation of basophils sensitised with this pooled serum displayed a characteristic bell-shaped curve over a wide range of allergen concentrations ranging from 10 µg/mL to 1 pg/mL, with an optimum at 100 pg/mL. An example for such dose-response curve from 1 ng/mL to 10 fg/mL is shown in Figure 3 (see Figure S3 in Supplementary data for higher concentration range).

Higher concentrations of human serum can display strong cytotoxic activities towards RBL cells, requiring the serum to be sufficiently diluted [35]. This dilution however also leads to dilution of the IgE present in the serum, potentially limiting sensitivity of the assay by reducing efficiency of sensitisation, particularly when using the traditional beta-hexosaminidase enzymatic assay for detection of degranulation and sera with low IgE titres [36].

Therefore in order to test the potential cytotoxic activity of the Par j 2-specific serum pool with and without anti-viral treatment the RS-ATL8 cells were sensitized overnight with different serum dilutions, challenging the cells with the previously determined optimal 100 pg/mL Par j 2 concentration and measuring luciferase activity after 4 hours. As shown in Figure 4, the highest concentration of untreated serum (10-fold dilution) reduced the measured luciferase activity by approximately two thirds compared with the optimal 50-fold dilution, which can be ascribed to the well documented cytotoxic effects of some human sera on RBL cells. The anti-viral treatment completely abrogates the luminescent response at higher serum concentrations but can be used efficiently in dilutions higher than 1∶100.

**Figure 5:**
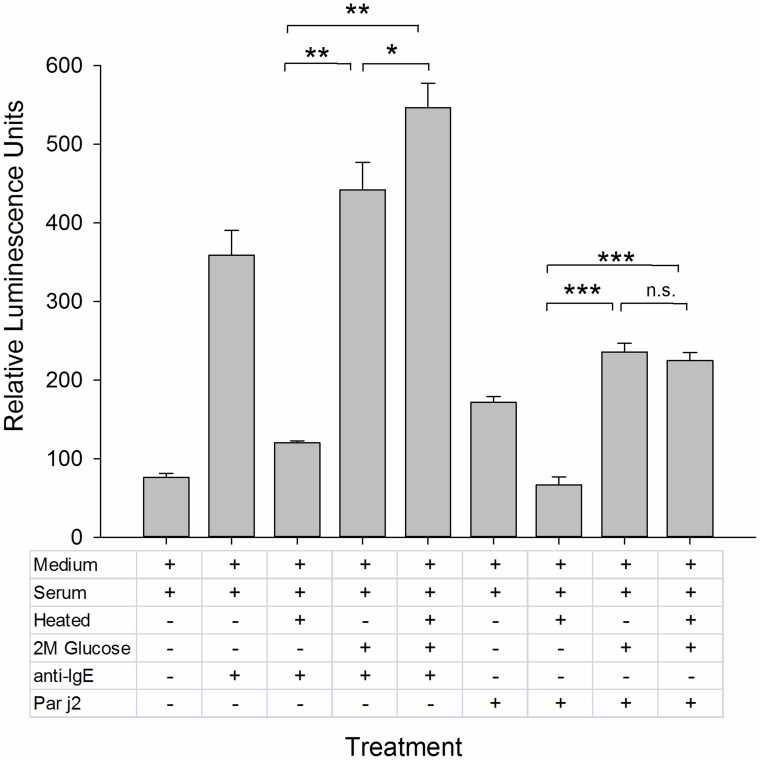
Protective effect of 2M Glucose on the ability of IgE to bind to the high affinity receptor after heating at 56°C for 30 min. RS ATL8 cells seeded at a density of 50.000 cells per well were sensitised with a 50-fold dilution of Par j 2 serum pool, which had been left unheated or heated either in the presence or in the absence of 2M Glucose. After overnight incubation with the sera, cells were stimulated with optimal concentrations of anti-IgE (1 µg/mL) or Par j 2 recombinant allergen (100 pg/mL) and luciferase production measured after 4 hours. ; *: p<0.05; **: *p*<0.01; ***: p<0.001; n.s.: not significant (Student t-test).

We assessed whether it is possible to heat sera in the presence of 2M glucose or 1M MgSO_4_, conditions which were shown by Binaghi [37] to prevent denaturation of IgE, without affecting successful inactivation of complement. The results are shown in Figure 5. Heating of serum in the presence of 2M glucose resulted in complete protection of IgE as judged by its ability to sensitise RS ATL8 cells and their ability to produce luciferase upon stimulation with anti-IgE or recombinant Par j 2 allergen.

**Figure 6:**
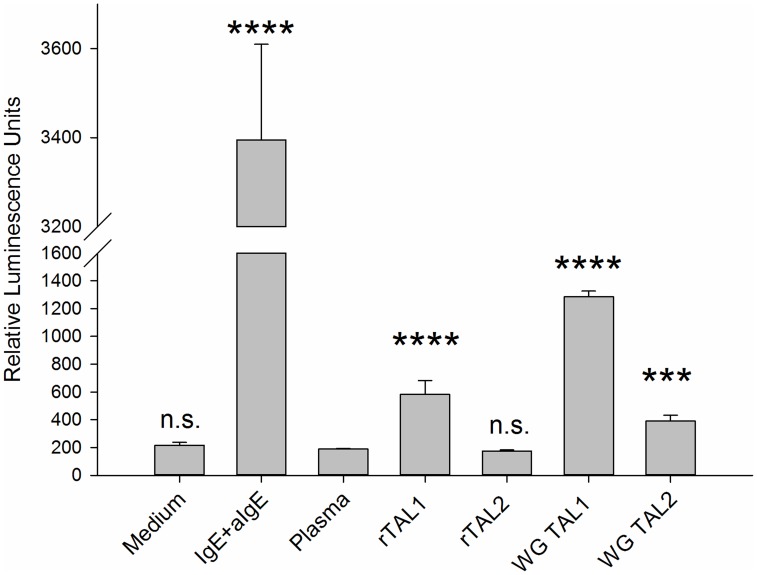
Luminescence data of RS-ATL8 assay demonstrating the allergenicity of SmTAL-1. Recombinant SmTAL1 and SmTAL2 were expressed in *E. coli* (rTAL1, rTAL2; both 10 ng/ml) and in WGL (WG TAL1, WG TAL2; diluted 1∶10^4^). Cells were sensitized for 16 hours with pooled, virally inactivated plasma from individuals living in a *S. mansoni* endemic area in Uganda (diluted 1∶100) and stimulated with the recombinant antigens. The experiment included the negative controls: unsensitized and unstimulated cells (medium), cells incubated only with treated plasma diluted 1∶100 v/v (serum) and the positive control (IgE+aIgE, both 1 µg/mL). Data are mean +SD of the readings of three separate wells. Multiplicity adjusted p-values were obtained by ANOVA followed by Dunnett's test for each condition compared with plasma only control. ****: *p*<0.0001; ***: p<0.001; n.s.: not significant.

**Figure 7:**
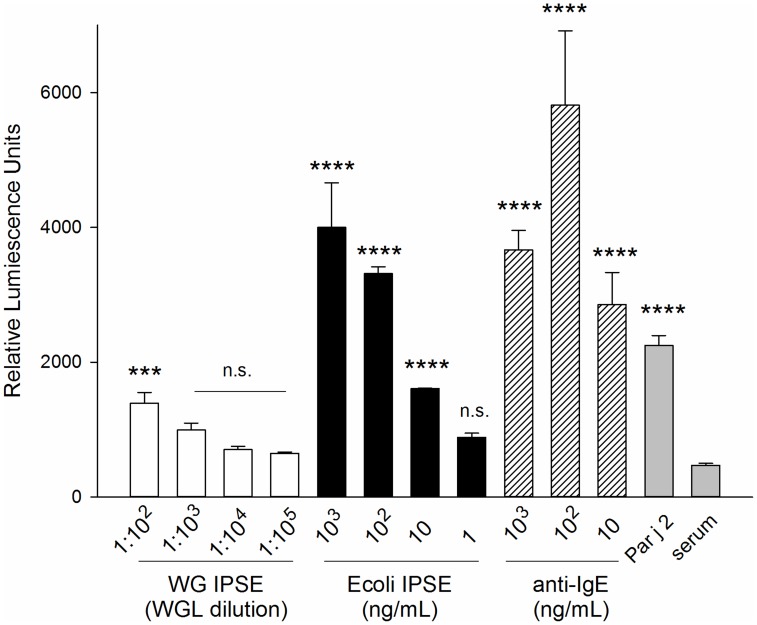
Activation of RS-ATL8 reporter system by IgE binding factor IPSE/alpha-1 expressed in wheat germ (white bars) and *E. coli* (black bars). All cells (except IgE only) were sensitized for 16 hours with pooled serum from *P. judaica* allergic individuals diluted 1∶50 (v/v). The experiment included positive controls: sensitized with 1 µg/mL IgE and stimulated with the indicated amounts of polyclonal anti-human IgE (hatched bars); stimulated with Par j 2 (100 pg/ml; grey bar), as well as the negative controls (grey bas): serum sensitized/unstimulated cells (serum only). Data are mean ±SD of the readings of three separate wells. Multiplicity adjusted p-values were obtained by ANOVA followed by Dunnett's test for each condition compared with serum only control ****: *p*<0.0001; ***: p<0.001; n.s.: not significant.

**Figure 8:**
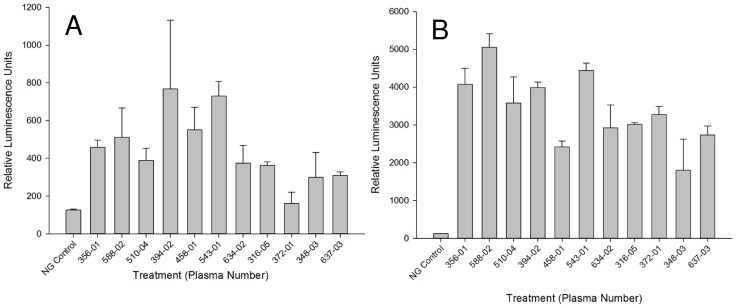
Screening of SmTAL-1 allergenicity with individual infected individuals' plasma. A and B) RS-ATL8 cells were sensitised for 16 hours with different unpooled plasma from *S. mansoni* infected individuals with known moderate to high SmTAL1-specific IgE titres determined by ELISA. After a wash, cells were challenged with 1/1000 diluted SmTAL-1 wheat germ lysate translation mixture in (A) or 1 µg/ml anti-IgE (B). Luminescence was measured 4 hours after stimulation. Shown is one experiment as mean of triplicate +/− SD.

Our next step was to test the ability of the reporter cell line sensitized with a serum pool from *S. mansoni*-infected individuals virally inactivated with Tween-80 to report engagement of the IgE receptor when stimulated with SmTAL-1 and SmTAL-2 produced in wheat germ lysates. A sensitisation to wheat allergens as a source of activation could be categorically ruled out as a source of error as activation only occurred in the presence of translated allergen but not with wheat germ lysate controls.

Figure 6 shows that stimulation of cells sensitized with a serum pool from *S. mansoni*-infected individuals with an optimal dilution of SmTAL-1 results in marked elevation of luciferase production, in contrast to stimulation with SmTAL-2. Interestingly, antigen expressed in wheat germ lysate resulted in stronger activation of the reporter system compared with the *E. coli* expressed equivalent, despite using optimal concentration from full dilution curves (Supplementary data Figure S4).

As many secretory proteins will rely on correct folding and formation of intra- or intermolecular disulphide bridges, which may affect their recognition by specific IgE, it is important to assess the ability of the wheat germ expression system to produce correctly folded parasitic antigens.

Assessing the ability of this particular protein to activate RS-ATL8 in this manner would provide further validation of the luciferase system to report IgE dependent activation events, while at the same time informing of the ability of the wheat germ lysate to produce disulphide-bridged homodimers. We therefore assessed the basophil activating properties of IPSE/alpha-1, an IgE-binding factor from *S. mansoni* which is known to rely on dimeric structure for its biological activity [33]. Figure 7 shows a comparison of the ability of wheat germ expressed IPSE/alpha-1 with the same protein expressed and refolded in *E. coli* to induce RS-ATL8 activation.

Both forms of IPSE/alpha-1 were able to dose-dependently induce reporter gene expression. However the effect was much more prominent with the bacterially-expressed refolded recombinant protein, which induced luciferase to an extent similar to the positive controls IgE/anti-IgE and Par j 2-specific serum/Par j 2. The luciferase induction by wheat germ-derived IPSE/alpha-1 was modest, suggesting that it is mainly present in its monomeric form, while the *E. coli* recombinant protein occurs as a mixture of monomers and dimers after refolding [33], and we have previously shown that basophil activation requires IPSE/alpha-1 in its dimeric structure [34].

**Figure 9:**
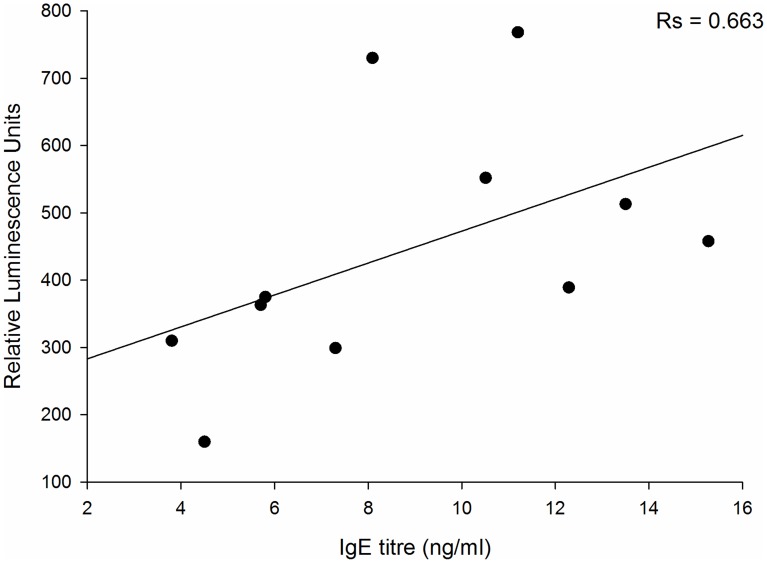
Correlation between the IgE titre measured in each plasma sample and the intensity of luciferase induction after stimulation with SmTAL-1. The Spearman rank correlation coefficient was R_s_ = 0.663 (p = 0.035, two-sided).

As specific IgE titres will vary between individuals, we assessed the ability of 11 individual sera from infected individuals (ranging from 3.8 to 15.27 ng/mL SmTAL-1 specific IgE, as determined by ELISA) to sensitise the RS ATL8 cell line. The sera ranged from RAST class 2 (moderate IgE, 07.70–3.49 IU/mL or 1.68–8.39 ng/mL) to RAST class 3 (high IgE, 3.50–17.49 IU/mL or 8.40–41.97 ng/mL). As can be seen from Figure 8A, all sera except serum 372-01 gave a positive response upon stimulation with an optimal concentration of SmTAL-1, showing that the RS ATL8 assessment works not only with sera with high levels of allergen-specific IgE, but also with moderate specific IgE levels. All sera gave vigorous responses upon polyclonal stimulation with 1 µg/mL anti-IgE (Fig. 8 B). Non-parametric analysis (Spearman rank correlation test) indicated a statistically significant positive correlation (Fig. 9) between levels of SmTAL-1-specific IgE and the amount of luciferase produced 4 hours after stimulation.

## Discussion

Our aim was to establish a workflow which is suitable for medium- to high-throughput screening of potential allergenicity of *S. mansoni* antigens and includes a set of three steps for stringent quality controls. The first two were implemented at the DNA level (correct size of the PCR amplicon and correct sequence after cloning into expression vector), while the third (successful translation and appropriate protein size by incorporation of fluorescently-tagged lysine) was at the protein level. We chose to incorporate fluorescently labelled Lys in a separate aliquot as this fluorophore may potentially affect the antigenicity or allergenicity of the *in vitro*-translated product.

Wheat germ lysate is a complex biological mixture and as such it has the potential to interfere with the cellular readout. In particular, the lectin wheat germ agglutinin (WGA) may play a critical role in the context of IgE receptor cross-linking. WGA is a 18 kDa lectin contained in wheat germ [38] which naturally occurs as a 36 kDa homodimer in which the two chains are linked with 16 disulfide bonds [39]. WGA is specific for *N*-acetyl-D-glucosamine and the chitin oligomers chitobiose and chitotriose [39]. WGA has been shown to inhibit RBL cell activation by engaging the high affinity IgE receptor FcεRI independently of its occupancy by IgE, leading to its down regulation and inhibition of allergen-induced signal transduction [40]. In contrast, in human peripheral blood basophils, WGA can lead to basophil activation and cytokine induction (IL-4 and IL-13) by cross-linking FcεRI-bound IgE via its carbohydrate side chains [41]. In line with this finding, the addition of undiluted wheat germ lysate used in this study resulted in non-specific, high luciferase signals. The WGA can be removed from the lysate by incubation with chitin beads either prior to addition of plasmid, or by the purification of the *in vitro* translated antigen via its incorporated His-Tag and immobilized metal-ion affinity chromatography. However, additional steps are to be avoided in a high-throughput procedure as they increase the overall cost, duration and introduce additional sources of errors. Due to the well-known bell-shaped curve of basophil activation and the high yield of the chosen *in vitro* translation system, the wheat germ lysate containing the translated parasitic protein has to be diluted 1000-fold to reach the concentration range around 100 pg/mL in which stimulation is optimal (as demonstrated by the use of Par j 2 allergen and matching sera in Figure 3). With this dilution, there is no inhibition of IgE receptor crosslinking or basophil activation by WGA. This also means that only small volumes of wheat germ lysate are needed for repeated testing, reducing the overall cost of a high throughput operation.

Thus while the potential disadvantages of choosing wheat germ lysate for translation (such as the lack of glycosylation) are not relevant for this assay when assessing anti-peptide IgE, there are multiple advantages. This system is amenable to high-throughput protein synthesis because it can produce sufficiently large amounts of properly folded proteins, and it bypasses many time-consuming steps of conventional expression systems. It also allows expression of proteins that are toxic to their host organism chosen for expression. Wheat germ lysates, in contrast to e.g. *E. coli* lysates, have very low endogenous mRNA levels, and most of the newly translated protein is thus of parasite (or other target) rather than plant origin. This allows for very efficient incorporation of non-radioactive labels for detection or other purposes. A limitation of the wheat germ system is the inability to provide a non-reducing environment together with all necessary compartmentalised components for disulphide bridge formation. This is demonstrated by our results obtained for IPSE/alpha-1. IPSE/alpha-1 is a secretory protein produced exclusively by *S. mansoni* egg stage [42], which naturally occurs as a homodimer with three intramolecular disulphide bridges and one intermolecular bridge formed by the most C-terminal cysteine in position C132 [33]. We have previously shown that this homodimeric molecule is able to activate human basophils by binding IgE molecules [43], and that IgE-dependent human basophil activation is dependent on its dimerization status [34]. However this limitation does not appear to affect this molecule's antigenicity (Fig. 2).

Using a humanised rat basophil cell line also has several advantages [22], [23]. It is easier to obtain than e.g. human peripheral blood basophils, which have been notoriously difficult to purify until recently [44], and are still difficult to obtain in sufficient amounts despite these advances due to their rarity. The cells are sensitized only with the desired sera and do not require difficult IgE stripping protocols [45]. Furthermore, known potential issues such as non-responder status [46] due to down regulation of key signalling molecules such as spleen tyrosine kinase (Syk) caused by chronic exposure to low level of allergens [47], which therefore might also be occurring in helminth infection, are avoided. Non-responder status is an issue we came across in a subset of individuals when assessing peripheral blood basophil sensitization status in a cohort experimentally infected with a single dose of ten *Necator americanus* infective stage larvae [21], and could lead to false negative results. Also as human IgG is not thought to bind to rat immunoglobulin receptors, and the rat high affinity IgE receptor does not bind human IgE [48], [49], there is no potential for confounding factors which could mask the potential allergenicity of the studied antigens, such as competing IgG4 [50], [51], inhibitory co-crosslinking of FcεRIα and FcγRIIB [52], or activation due to IgG-IgE immune complexes [53], as these factors are removed by washing the reporter cell line prior to allergen stimulation. Finally, using the NFAT luciferase reporter for detection of activation, rather than the traditionally used β-hexosaminidase biochemical assay, results in considerably increased sensitivity. The ability of the RS-ATL8 to detect sensitisation with less than 100 pg IgE (Supplementary data S2) compares favourably with previously reported limit of 10 ng/ml upon polyclonal stimulation with an anti-IgE antibody using β-hexosaminidase activity for detection [54]. We have previously assessed alternative methods of measuring activation induced by IgE crosslinking in RBL cells (traditional beta-hexosaminidase assays, Annexin V measurements, CD63 and CD107a levels by FACS, Calcium Influx using Oregon Green or Alexa488 BAPTA-1, as well Lucifer yellow uptake, but none of these methods worked or offered any advantage over luciferase measurements.

Serum samples which had undergone anti-viral treatment with a mixture of detergents had to be diluted at least 100-fold, as higher concentrations of detergents led to complete destruction of cells as assessed by microscopy and the complete lack of luciferase induction. Tween-80 is the major constituent in the viral inactivation detergent used here and has a critical micellar concentration (CMC) of 0.012 mM [55] which lies precisely in between the concentration of Tween-80 in the 50-fold and 100-fold dilutions of treated sera. Thus a 100-fold dilution of sera will work to reduce serum cytotoxicity for both untreated and virally inactivated sera. As previously shown, serum cytotoxicity in this system is probably in part due to complement activation, as heating the serum at 56°C for 30 min reduced its cytotoxicity [35]. This treatment however irreversibly denatures IgE, abrogating its binding to the high affinity IgE receptor [56]. Our results demonstrate that 2M glucose treatment might represent a suitable way of inactivating complement in sera without leading to loss of IgE functionality. However, subsequent experiments with glucose-treated sera clearly showed that these also had to be diluted 20–50-fold to avoid deleterious effects of the high glucose concentration in the cellular assay, or required dialysis-based methods. As this would complicate the workflow unnecessarily, we did not pursue these attempts, and used serum dilutions of 1∶50 with virally non-inactivated sera or 1∶100 to 1∶200 with virally inactivated, detergent-treated sera. The work described in Figures 3–5 and S2, was performed using a well-characterised (ISAC UniCAP, clinical history) serum pool from *P. judaica* allergic individuals, rather than sera from parasite-infected individuals and helminthic allergens. This was mainly due to the unavailability of large amounts of infection serum required for such studies, but also as it allowed us to validate the technology against the current gold standard for specific IgE determination. The results are equally relevant for IgE/allergen combinations studied in a tropical parasite infection context, as the underlying mechanisms of sensitisation, cellular activation and allergenicity are fundamentally the same [57].

The SmTAL proteins are a family of 13 closely related allergen-like molecules. Of these SmTAL1 and 2 have the greatest similarity in amino acid sequence (48% identity). However, in populations from endemic areas, SmTAL1 is reported to be the dominant IgE-inducing antigen whilst an IgE response to SmTAL2 is rare [26], [31]. It has been proposed that this is because the response to egg antigen – SmTAL2 – is desensitized by continuous exposure (as eggs die in the host tissues every day); whilst internal adult worm antigen SmTAL1 is only exposed infrequently when adult parasites die [26]. An interesting observation was made when comparing the allergenicity of SmTAL-1 and SmTAL-2 between the bacterial recombinant forms and their wheat germ expressed counterparts. We carried out full titration curves with all four but found that in both cases the WG-expressed form induced significantly higher reporter cell activation than the *E. coli* expressed SmTALs (Supplementary data S4). The reasons for this difference are not clear, but would appear to rule out that LPS contamination of bacterially expressed recombinant allergens could be a source of basophil activation in the used assay.

Taken together, this method offers a robust way for assessing potential allergenicity of *S. mansoni* (or any other parasite) in a format suitable for high-throughput analysis. The novelty of the method presented here lies in the combination of a fast cell-free expression system and an equally fast reporter system which allows expression of candidate allergens in a few hours and detection of activation within three hours, all up-scalable to high-throughput format. This method can be used as an additional safety test when assessing potential vaccine candidates. Perhaps more importantly, when used at the whole genome level, it could be used to unravel the entire allergome of *S. mansoni* and other medically important parasites. Ultimately this could lead to a better understanding of the basis of allergenicity, and in combination with additional cellular studies, to a better understanding of the relationship between parasite-specific IgE and host protection mechanisms at the molecular level [57].

## Supporting Information

Figure S1 In gel detection of ten different *S. mansoni* antigens (DA18 to DA30, from right to left) expressed *in vitro* using wheat germ lysate. Success of translation was monitored by incorporation of BODIPY-labelled fluorescent Lysine during translation in separate aliquots. Samples were run on 4–20% SDS-PAGE gradient gels under reducing conditions and imaged in a Fujifilm LAS-4000. The left lane (ctrl) includes the wheat germ lysate control without template DNA, indicating fluorescent components produced during *in vitro* translation from endogenous mRNA. The expected molecular weights were: DA30 (14.3.3; Smp_009760): 29.3 kDa; DA29 (IPSE alpha-1; Smp_112110): 13.3 kDa; DA27: (Major Egg antigen; Smp_049300.3): 40.3 kDa; DA26 (Haemoglobinase; Smp_075800): 47.1 kDa; DA25 (Troponin T, Smp_179810): 37.4 kDa; DA23 (triosephosphate isomerase, Smp_003990): 29.0 kDa; DA22 (Sm14 fatty acid-binding protein isoform T20, Smp_095360.3): 11.9 kDa; DA20 (ornithine aminotransferase, Smp_000660): 48.5 kDa; DA-19 (ngng-dimethylarginine dimethylamino-hydrolase, Smp_052560 17.0 kDa); DA-18 (GST class mu; SM26/2 antigen, Smp_102070): 23.5 kDa. Apparent MW may vary from the values described in the literature due to lack of glycosylation, the presence of a (His)_6_Val tag added at the C-term of each protein, or the removal of an N-terminal signal peptide (see Table S3 in Supplementary data for additional information).
(TIF)

Figure S2 IgE detection limit of RS-ATL8 reporter cell line. 100,000 RS-ATL8 cells were sensitized overnight with different concentrations of monoclonal IgE (ranging from 5000 to 0.1 ng/mL) in a 100 µl volume. Cells were then stimulated with 1 µg/mL polyclonal anti-IgE. Results are mean +/− s.d. of an experiment performed in quadruplicate determination. T-test results indicate p<0.001 significance for all conditions compared with cell sensitized with 1 µg/mL IgE, but not stimulated (IgE only).
(TIF)

Figure S3 Optimal concentration and minimal concentration of detectable allergen was determined by sensitising RS-ATL-8 cells with the pooled serum (1∶50 dilution) from individuals with a monospecific IgE response to the Par j 2 allergen and challenged with recombinant Par j 2, serially diluted from 10 µg/mL to 10 pg/mL in 1∶10 dilution increments. Data are mean ±SD of the readings of three separate wells.
(TIF)

Figure S4 Activation of RS ATL-8 reporter cell line by serial dilutions of SmTAL1 and SmTAL2 expressed in *E. coli* (rTAL1, rTAL2) or using WGL (WG TAL-1, WG-TAL2) as well as negative (medium only, serum only) and positive polyclonal activation (IgE+anti-IgE) control.
(TIF)

Table S1 Details of S. mansoni infected individual plasma samples used in this study (MUG6 *S.mansoni* infected human sera). 10×30 µl post-treatment with praziquantel (PZQ) plasma samples, which have been virally inactivated as described by Poulsen and Sørensen [32] were pooled in equal amounts. Samples were classified as high vs medium titre groups according to RAST class equivalence. Epg = eggs per gram faeces.
(DOCX)

Table S2 Characterisation of Par j 2 –specific serum pool by ISAC 103.
(XLS)

Table S3 Details of genes used in this work. Most proteins have a His-Tag and a terminal Valine added at the C-term. Proteins with a predicted or known classical signal peptide (SS for signal sequence) had the signal removed during cloning.
(XLS)
