# Correction: Use of Humanised Rat Basophilic Leukaemia Cell Line RS-ATL8 for the Assessment of Allergenicity of *Schistosoma mansoni* Proteins

**DOI:** 10.1371/journal.pntd.0003331

**Published:** 2014-10-22

**Authors:** The PLOS Neglected Tropical Diseases Staff

## Notice of Republication

This article was republished on October 1^st^, 2014. The author inadvertently uploaded the incorrect versions of Figures 3-7, 9 and Supporting Information files S2, S3 and S4 upon resubmission. PLOS apologizes for not catching this error prior to publication. Please download this article again to view the correct version. The originally published, uncorrected article and the republished, corrected article are provided here for reference.

## Supporting Information

File S1 Originally published, uncorrected article.
(PDF)

File S2 Republished corrected article.
(PDF)
