# Supplementary figures and images for: Use of Humanised Rat Basophilic Leukaemia Cell Line RS-ATL8 for the Assessment of Allergenicity of Schistosoma mansoni Proteins
Source: PLoS Negl Trop Dis. 2014 Sep 25;8(9):e3124. doi: 10.1371/journal.pntd.0003124 (PMC4177753; doi:10.1371/journal.pntd.0003124)

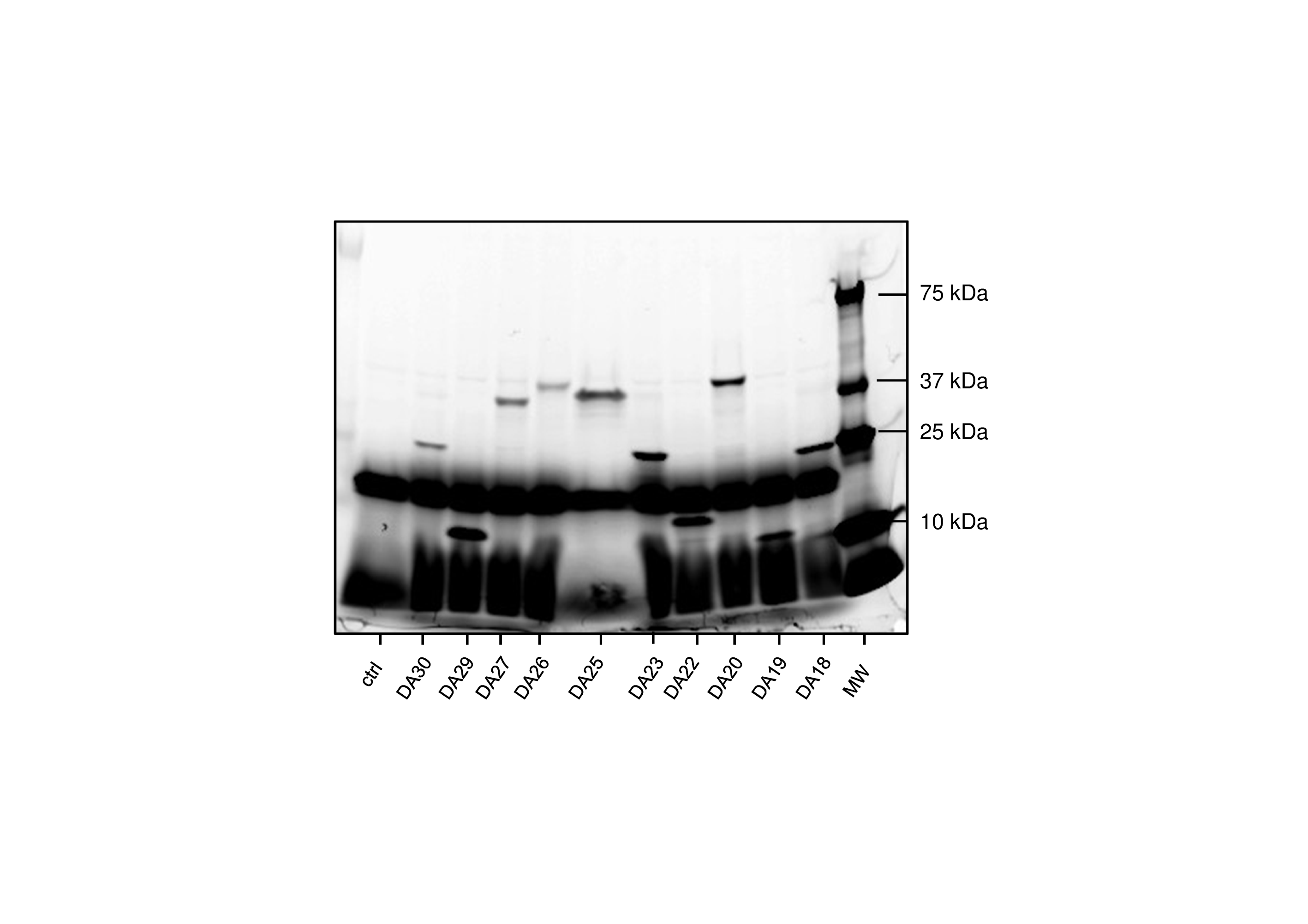

Supplement: Figure S1 — In gel detection of ten different S. mansoni antigens (DA18 to DA30, from right to left) expressed in vitro using wheat germ lysate. Success of translation was monitored by incorporation of BODIPY-labelled fluorescent Lysine during translation in separate aliquots. Samples were run on 4–20% SDS-PAGE gradient gels under reducing conditions and imaged in a Fujifilm LAS-4000. The left lane (ctrl) includes the wheat germ lysate control without template DNA, indicating fluorescent components produced during in vitro translation from endogenous mRNA. The expected molecular weights were: DA30 (14.3.3; Smp_009760): 29.3 kDa; DA29 (IPSE alpha-1; Smp_112110): 13.3 kDa; DA27: (Major Egg antigen; Smp_049300.3): 40.3 kDa; DA26 (Haemoglobinase; Smp_075800): 47.1 kDa; DA25 (Troponin T, Smp_179810): 37.4 kDa; DA23 (triosephosphate isomerase, Smp_003990): 29.0 kDa; DA22 (Sm14 fatty acid-binding protein isoform T20, Smp_095360.3): 11.9 kDa; DA20 (ornithine aminotransferase, Smp_000660): 48.5 kDa; DA-19 (ngng-dimethylarginine dimethylamino-hydrolase, Smp_052560 17.0 kDa); DA-18 (GST class mu; SM26/2 antigen, Smp_102070): 23.5 kDa. Apparent MW may vary from the values described in the literature due to lack of glycosylation, the presence of a (His)6Val tag added at the C-term of each protein, or the removal of an N-terminal signal peptide (see Table S3 in Supplementary data for additional information). (TIF) [file pntd.0003124.s001.tif]

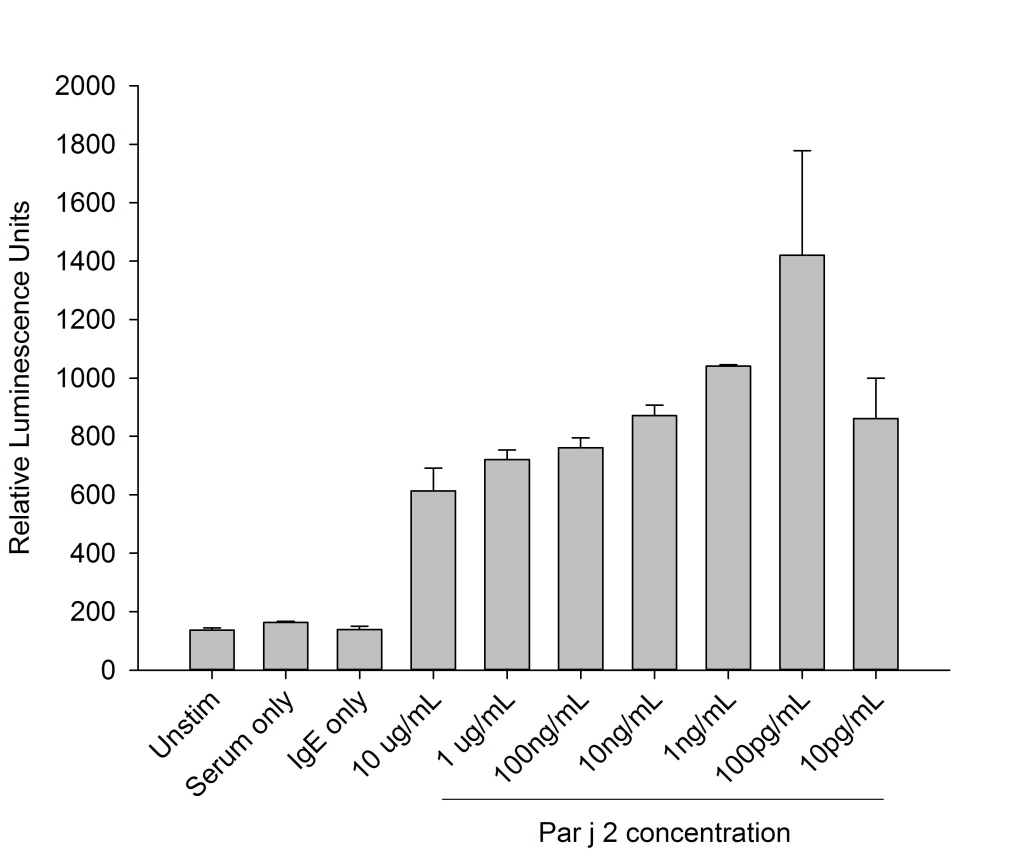

Supplement: Figure S3 — Optimal concentration and minimal concentration of detectable allergen was determined by sensitising RS-ATL-8 cells with the pooled serum (1∶50 dilution) from individuals with a monospecific IgE response to the Par j 2 allergen and challenged with recombinant Par j 2, serially diluted from 10 µg/mL to 10 pg/mL in 1∶10 dilution increments. Data are mean ±SD of the readings of three separate wells. (TIF) [file pntd.0003124.s003.tif]
