# Supplementary material for: Use of Humanised Rat Basophilic Leukaemia Cell Line RS-ATL8 for the Assessment of Allergenicity of Schistosoma mansoni Proteins
Source: PLoS Negl Trop Dis. 2014 Sep 25;8(9):e3124. doi: 10.1371/journal.pntd.0003124 (PMC4177753; doi:10.1371/journal.pntd.0003124)
Supplement: Table S1 — Details of S. mansoni infected individual plasma samples used in this study (MUG6 S.mansoni infected human sera). 10×30 µl post-treatment with praziquantel (PZQ) plasma samples, which have been virally inactivated as described by Poulsen and Sørensen [32] were pooled in equal amounts. Samples were classified as high vs medium titre groups according to RAST class equivalence. Epg = eggs per gram faeces. (DOCX) [file pntd.0003124.s005.docx]

| Patient ID | Age | Infection  Pre PZQ epg | Post rx SmTAL1_IgE (ng/ml) | Titre Group |
| --- | --- | --- | --- | --- |
| 356_01 | 25 | 4583 | 15.27 | High |
| 588_02 | 25 | 577 | 13.5 |  |
| 510_04 | 11 | 593 | 12.29 |  |
| 394_02 | 13 | 1440 | 11.2 |  |
| 458_01 | 26 | 897 | 10.51 |  |
| 543_01 | 26 | 1090 | 8.09 |  |
|  | | | | |
| 316_05 | 15 | 1163 | 5.7 | Medium |
| 348_03 | 17 | 3173 | 7.3 |  |
| 372_01 | 37 | 966 | 4.5 |  |
| 637_03 | 20 | 863 | 3.8 |  |
